# The emerging role of NG2 in pediatric diffuse intrinsic pontine glioma

**DOI:** 10.18632/oncotarget.3716

**Published:** 2015-03-30

**Authors:** Sridevi Yadavilli, Joseph Scafidi, Oren J. Becher, Amanda M. Saratsis, Rebecca L. Hiner, Madhuri Kambhampati, Santi Mariarita, Tobey J. MacDonald, Kari-Elise Codispoti, Suresh N. Magge, Jyoti K. Jaiswal, Roger J. Packer, Javad Nazarian

**Affiliations:** ^1^ Research Center for Genetic Medicine, Children's National Health System, Washington, DC, USA; ^2^ Department of Neurology and Center for Neuroscience Research, Children's National Health System, Washington, DC, USA; ^3^ Department of Pediatrics and Pathology, Preston Robert Tisch Brain Tumor Center, Duke University Medical Center, Durham, NC, USA; ^4^ Division of Neurosurgery, Ann & Robert H. Lurie Children's Hospital of Chicago, Chicago, IL, USA; ^5^ Human Oncology and Pathogenesis Program, Memorial Sloan-Kettering Cancer Center, NY, USA; ^6^ Department of Pathology and Lab Medicine, The Children's Hospital of Philadelphia, Philadelphia, PA, USA; ^7^ Department of Pediatrics, Emory University School of Medicine, Atlanta, GA, USA; ^8^ Department of Pathology, Children's National Health System, Washington, DC, USA; ^9^ Division of Neurosurgery, Children's National Health System, Washington, DC, USA; ^10^ Department of Integrative Systems Biology, George Washington University School of Medicine and Health Sciences, Washington, DC, USA; ^11^ Brain Tumor Institute, Center for Neuroscience and Behavioral Medicine, Children's National Health System, Washington, DC, USA

**Keywords:** DIPG, NG2, PDGF, histone 3, glioma

## Abstract

Diffuse intrinsic pontine gliomas (DIPGs) have a dismal prognosis and are poorly understood brain cancers. Receptor tyrosine kinases stabilized by neuron-glial antigen 2 (NG2) protein are known to induce gliomagenesis. Here, we investigated NG2 expression in a cohort of DIPG specimens (n= 50). We demonstrate NG2 expression in the majority of DIPG specimens tested and determine that tumors harboring histone 3.3 mutation express the highest NG2 levels. We further demonstrate that microRNA 129-2 (miR129-2) is downregulated and hypermethylated in human DIPGs, resulting in the increased expression of NG2. Treatment with 5-Azacytidine, a methyltransferase inhibitor, results in NG2 downregulation in DIPG primary tumor cells *in vitro*. NG2 expression is altered (symmetric segregation) in mitotic human DIPG and mouse tumor cells. These mitotic cells co-express oligodendrocyte (Olig2) and astrocyte (glial fibrillary acidic protein, GFAP) markers, indicating lack of terminal differentiation. NG2 knockdown retards cellular migration *in vitro,* while NG2 expressing neurospheres are highly tumorigenic *in vivo,* resulting in rapid growth of pontine tumors. NG2 expression is targetable *in vivo* using miR129-2 indicating a potential avenue for therapeutic interventions. This data implicates NG2 as a molecule of interest in DIPGs especially those with H3.3 mutation.

## INTRODUCTION

Pediatric brainstem gliomas (BSGs) account for 15% of all brain tumors in children [1]. Diffuse intrinsic pontine gliomas (DIPGs) are high grade tumors of pons with the peak age of onset of 6-7 years [2]. Children diagnosed with DIPG have less than 90% chance of survival within two years of diagnosis [3, 4]. A pLys27Met (K27M) driver mutation in the *H3F3A* gene of histone 3 variant 3 (H3.3) and *HIST1H3B* (H3.1) was recently correlated to a subgroup of DIPG patients with distinct clinical and biological characteristics [5]. Other genomic aberrations of DIPGs include p53 mutations and amplification of tyrosine receptor kinase/Ras/phosphatidylinositol 3-kinase signaling pathways including platelet derived growth factor receptor alpha (PDGFRα) [6]. Our group and others have reported the involvement of Hedgehog (Hh) signaling pathway in a subset of DIPGs [7, 8]. Modulation of Hh and tyrosine kinase receptors may alter the self-renewal properties of DIPG cells by targeting the self-renewing cancer stem cells (CSC). Receptor tyrosine kinases including PDGFRα are stabilized by the transmembrane protein NG2, also known as chondroitin sulfate proteoglycan 4 (CSPG4) [9]. NG2^+^ cells that often co-express PDGFRα and Olig-2 are present in adult gliomas [10-12], where NG2 contributes to the neoplastic transformation of glioma precursor cells [13]. NG2 segregation in dividing oligodendrocytes plays an important role in terminal differentiation and self-renewal properties of these cells [13]. Specifically, in non-neoplastic tissue, NG2 expression is limited to only one daughter cell. In contrast, in malignant gliomas, NG2 is symmetrically expressed in both daughter cells resulting in active expression of other growth factor receptors including epidermal growth factor receptor (EGFR) [13]. Despite the potential role of NG2 in EGFR-mediated neoplasm, NG2 expression has not been previously established in pediatric gliomas. We have recently reported elevated mRNA expression of NG2 in a large percentage of pediatric DIPGs [8].

In this study, we determined that NG2 expression is prominent in a majority of our DIPG cohort (n=34) as well as *in vitro* and *in vivo* models of DIPG. Our study is the first to demonstrate that: i) NG2 expression is associated with DIPG; ii) NG2 expression is symmetric in mitotic cells resulting in uncommitted progenitors with CSC properties; iii) NG2 in DIPGs is regulated by miR129-2; and iv) NG2 expression can be targeted *in vivo* and *in vitro* using miR129-2. Orthotopic injection of NG2 expressing cells results in rapidly developing pontine tumors that co-express PDGFRα, PDGFRβ and Ki67. Identification of NG2 as a protein associated with DIPG may provide novel avenues for development of therapeutic targets to stop proliferation of this highly infiltrative cancer.

## RESULTS

### NG2 is upregulated in Human DIPG

**Figure 1:**
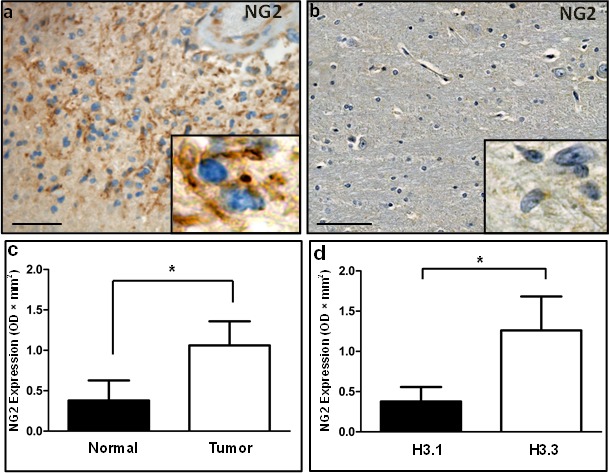
Immunohistochemical and western blotting analysis validates NG2 upregulation in human DIPG (**a**) In human tissue, diffuse expression of NG2 was observed in DIPG tumor localized to membrane (inset). (**b**) Adjacent healthy brainstem tissue. Scale bar: 100 μM. (**c**) Graphical representation of western blotting data of NG2 expression in 22 DIPG tumor and 16 adjacent normal specimens. GAPDH was used for protein loading normalization. Average NG2 expression was plotted after normalizing NG2 levels to GAPDH in each sample. *P* < 0.05 was considered significant. (**d**) Average of normalized NG2 expression levels in NG2 western blot samples with histone 3.1 K27M mutation or histone 3.3 K27M mutation. *P* < 0.05 was considered significant.

To investigate NG2 expression in DIPGs, we performed immunohistochemical (IHC) staining on formalin fixed paraffin embedded (FFPE) specimens from DIPG children obtained at postmortem. Histological studies by a neuropathologist indicated NG2 protein upregulation in tumor (Figure 1a), where NG2 was localized to the cell membrane as expected (Figure 1a, inset). NG2 expression was not detected in adjacent normal human brainstem (Figure 1b). To define the frequency of NG2 expression in DIPGs, we used a larger cohort (n = 50) of human specimens (Supplementary Table 1) for immunohistochemical (IHC) and Western blotting assays. IHC assays using formalin fixed specimens showed NG2 expression in 75% (9 of 12) of DIPGs, with variable expression levels localized to tumor cells (Supplementary Table 2). To quantify NG2 upregulation, protein extracts from frozen human DIPG specimens were used for Western blotting assays (Supplementary Figure 1). NG2 expression in protein extract from 38 human specimens (22 DIPG and 16 adjacent normal) validated NG2 expression in DIPGs [13 of 22 (60 %)] to varying degrees. Low NG2 expression was also detected in four adjacent normal tissue specimens (Supplementary Figure 1). This could be attributed to the presence of infiltrating glioma cells or normal NG2 expressing cells within normal brainstem (Supplementary Figure 1). NG2 expression was assessed using densitometry and found to be significant (average fold change = 3, *p* < 0.05) in tumor compared to normal specimens (Figure 1c).

Mutation of the *HIST1H3B and H3F3A* genes, encoding histones 3.1 (H3.1) and 3.3 (H3.3) respectively, have been implicated in pediatric DIPGs [5, 14, 15]. We inspected the correlation of NG2 expression with H3 mutations status in our DIPG cohort (n = 13). We found NG2 to be significantly (p = 0.035) upregulated in H3.3 mutant specimens when compared with specimens with H3.1 mutation (Figure 1d). No NG2 expression was detected in the only DIPG specimen with wild type histone 3 status.

### NG2 Expression is Upregulated in Preclinical DIPG Models

**Figure 2:**
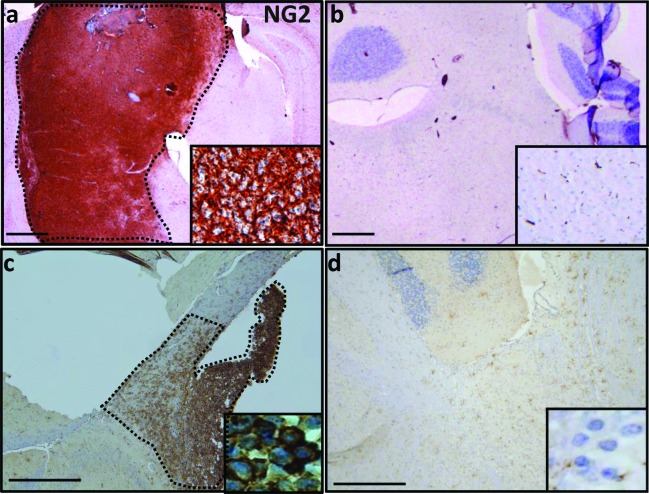
NG2 overexpression in PDGFB and PDGFB/H3.3 K27M/p53^−/−^ mouse models (**a**) In mouse, expression of NG2 in PDGFB mouse was limited to brainstem tumor (dotted area). (**b**) Sporadic expression of NG2 across brainstem and cerebellum of normal mouse. (**c**) Injection of NSG *SCID* mouse with mouse DIPG cells that are H3.3.K27 mutant and have PDGFB overexpression and p53 deletion resulted in overexpression of NG2 (dotted area). (**d**) In the adjacent normal brain tissue of NSG *SCID* mouse injected with PDGFB/H3.3.K27M/p53^−/−^ cells, NG2 overexpression was not detected. Insets are 40 × magnifications of the corresponding panel. Scale bar: 200 μM.

To test NG2 expression in preclinical DIPG murine models, we used IHC to assess NG2 expression in the PDGFB-Ink4a-ARF^−/−^ mouse (PDGFB model) [16] as well as mouse injected with glioma cells derived from PDGFB/p53^−/−^/H3.3K27M model [17]. We show significant upregulation of NG2 in both PDGFB (Figure 2a) and PDGFB/p53^−/−^/H3.3K27M (Figure 2c) DIPG mouse models. Low NG2 expression was detected in normal adjacent brainstem regions as well as the cerebellum (Figure 2b and 2d). High level (148 fold, *p* < 0.05) of NG2 was also detected by Western blot assays using tumor (n = 3) and normal (n = 3) brainstem specimens obtained from PDGFB mouse model or healthy controls (Supplementary Figure 2a). Furthermore, we tested NG2 expression in three primary human DIPG lines (SF8628, SUDIPGIV and SUDIPGVI) (Supplementary Figure 2b). All three primary cells showed NG2 expression as assessed by Western blotting assays.

### NG2 is Regulated by microRNA 129-2

**Figure 3:**
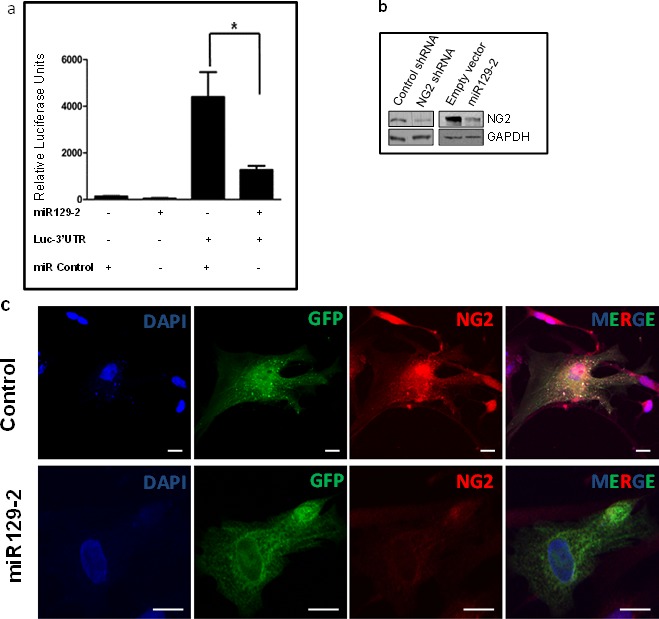

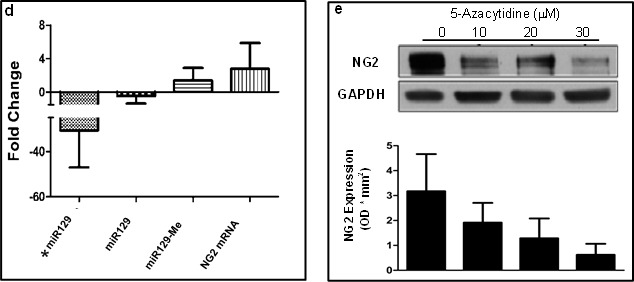
NG2 upregulation in DIPG is partially due to hypermethylation and downregulation of its regulatory microRNA, miR129-2 (**a**) Luciferase vector with 3′-UTR sequence of NG2 cloned downstream of the firefly luciferase gene (Luc-3′UTR) and plasmid vector with miR129-2 sequence were used. PDGFB mouse neurospheres were transfected in triplicates with luciferase and miR129-2 vectors, as shown, and luciferase expression was determined. Co-expression of Luc-3′UTR of NG2 and miR129-2 resulted in significant downregulation of luciferase expression as compared to cells transfected with luc-3′UTR and empty vector. *P* < 0.05 was considered significant. (**b**) PDGFB mouse neurospheres were transfected with miR129-2 or NG2-shRNA plasmids. Upregulation of either miR129-2 or NG2-shRNA resulted in NG2 downregulation *in vitro* when compared to empty vector or control shRNA treated cells. (**c**) Human DIPG cells, SF8628, were transduced with GFP expressing pCDH-control or pCDH-miR129-2 lentivirus. Immunofluorescence analysis by confocal microscopy revealed upregulation of miR129-2 (indicated by GFP) resulted in NG2 (red) downregulation *in vitro* when compared to control transduced cells. DAPI was used to stain nuclei. Scale bar = 5 μm. (**d**) Average expression of miR129-2 as assessed by RT-PCR (*miR129) or illumina expression chip assay (miR129), miR129 hypermethylation (miR129 me), and NG2 mRNA levels in human DIPG compared to adjacent normal. miR129-2 is shown to be downregulated in DIPG as assessed by RT-PCR and illumina chip assays. Hypermethylation of miR129-2 at four or more CpG sites corresponding to miR129-2 promoter were detected in 57% of DIPGs. As expected, downregulation of miR129-2 due to hypermethylation resulted in upregulation of NG2 mRNA as detected by NG2 mRNA using illumina platform. (**e**) To reverse miR129-2 hypermethylation, PDGFB mouse neurospheres were treated with 5-Azacytidine, a DNA methyl-transferase inhibitor drug. NG2 expression decreased in mouse tumor cells when treated with 10-30 μM 5-Azacytidine as compared to untreated cells. NG2 expression in each sample was normalized to corresponding GAPDH signal and average NG2 expression from three experiments was plotted.

To investigate potential molecular mechanism of NG2 regulation, we used Ingenuity Pathway Analysis (IPA) software, which generates biologic pathways based on published molecular interactions [8]. IPA analysis identified miR129-2 as a potential NG2 regulator. To validate NG2 as a target for miR129-2, we transfected PDGFB mouse neurospheres with a vector containing the 3′-UTR sequence of NG2 cloned downstream of the firefly luciferase gene (Luc-3′UTR) along with a plasmid vector containing miR129-2. We found that co-expression of Luc-3′UTR and miR129-2 results in significant downregulation of luciferase expression as compared to cells transfected with luc-3′UTR vector alone (Figure 3a). Moreover, transient transfection of PDGFB mouse tumor cells with NG2-targeting shRNA or miR129-2 expressing plasmid resulted in downregulation [(76% and 63%, respectively) as compared to control shRNA treated or empty vector treated cells] of NG2 protein as assessed by Western blot assay, validating the role of miR129-2 in regulation of NG2 expression (Figure 3b).

### miR129-2 Regulates NG2 in Human DIPGs

To validate miR129-2 mediated NG2 targeting in human DIPG, we used primary DIPG lines (SF8628). Human DIPG cells were transduced with pCDH-control or pCDH-miR129-2 lentivirus. Immunofluorescence analysis by confocal microscopy revealed upregulation of miR129-2 (indicated by GFP), which resulted in NG2 (red) downregulation *in vitro* when compared to control transduced cells (Figure 3c).

To investigate miR129-2 expression pattern in human DIPGs, we extracted total RNA from 7 tumor and 7 normal samples in our DIPG cohort and performed RT-PCR using miR129-2 primers (Figure 3d). We detected downregulation of miR129-2 in 85.7% (6 of 7) of DIPG tumors compared to normal tissue (average FC = −30.79, n=7 pairs). In five of these patients, RT-PCR generated miR129-2 expression levels corresponded with levels detected via whole transcriptome analysis of the same DIPG cohort (average FC = −2, n=7 pairs) [8]. We recently generated the comprehensive DNA methylation profiles of human DIPG specimens and their perilesional normal sections (n=9 pairs) [8]. To determine whether miR129-2 is hypermethylated in DIPGs, we inspected methylation patterns at 8 CpG loci corresponding to the miR129-2 promoter in DIPG specimens. We found overall hypermethylation in 66.6 % of specimens (6 of 9) with an overall average fold change in expression of 1.30 (tumor vs. normal, n=9 pairs, Figure 3d). Hypermethylation (at > 4 CpG sites) corresponded to decreased miR129-2 expression in 57% (4 of 7) of these patients (Figure 3d). Importantly, 71.4 % (5 of 7) of patients with downregulated miR129-2 expression also demonstrated NG2 upregulation at the gene (Figure 3d) or protein level in whole transcriptome and proteomic analysis.

### NG2 Expression is controlled *In Vitro* by Demethylating Drugs

Epigenetic silencing of miR129-2 is associated with cancer and has been shown to be reversible by demethylating drugs, including 5-Azacytidine (5-Aza) [18]. 5-Aza is a DNA methyl-transferase inhibitor that has also been used successfully in epigenetic modification of *SOX2* in adult malignant glioma cells [19]. We used PDGFB mouse cells and show that treatment with 5-Aza results in a significant downregulation of NG2 expression in these as compared to untreated cells (Figure 3e).

### NG2 Knockdown Decreases Cellular Migration *In Vitro*

In order to study the role of NG2 in cellular migration, we expanded PDGFB mouse BSG cells *in vitro* to form adherent monolayers. Scratch invasion assay, an established *in vitro* method that measures migration of adhered cells across the mechanically induced wound was performed [20], where cells were transfected with NG2-shRNA, miR129-2, or treated with demethylating drug 5-Aza (Supplementary Figure 3). Comparisons were done to cells treated with vehicle, control shRNA, or with empty vectors. We observed a significant reduction in NG2 expression that corresponds to decreased cell invasion in all treated cells compared to controls (Supplementary Fig 3). Specifically, treatment with NG2-shRNA, miR129-2 and 5-Aza resulted in 51% (*p* < 0.001), 51% (*p* < 0.001) and 82.5% (*p* < 0.001) cell invasion inhibition, respectively. Treatment with NG2-shRNA and miR129-2 resulted in a very similar reduction in cell invasion (51%), which may rule out an off-target effect of the two constructs.

### Mitotic DIPG Cells Exhibit Symmetric NG2 Expression Resulting in Co-Expression of Oligodendrocyte and Astrocyte Markers

**Figure 4:**
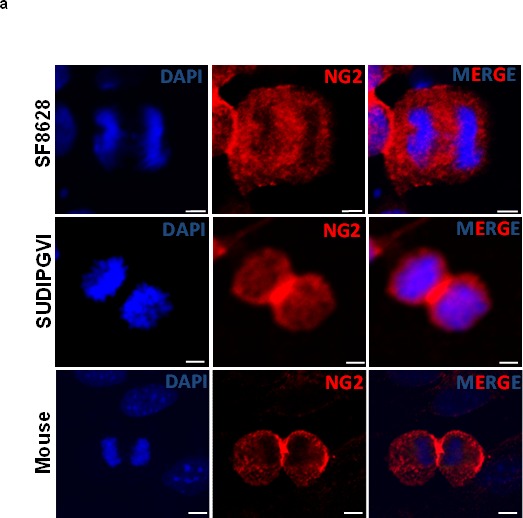

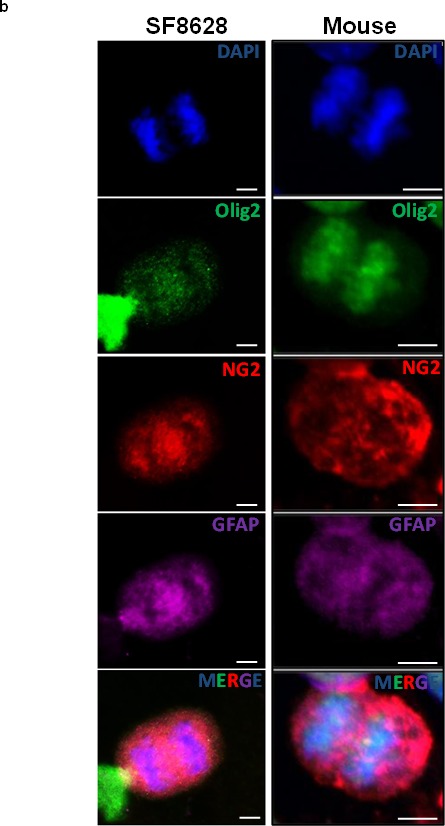
Mouse and human DIPG neurospheres exhibit symmetric NG2 expression *in vitro* and are immature stem cells co-expressing oligodendrocyte (Olig2) and astrocyte (GFAP) markers (**a**) PDGFB mouse tumor neurospheres (Mouse) and human primary DIPG cells (SF8628 and SUDIPGVI) were grown and stained using NG2 antibody (red) and DAPI (blue) nuclear staining. NG2 is equally expressed in dividing cells suggesting that its expression is defective (symmetric) in mitotic cells. Scale bar = 5 μm. (**b**) PDGFB mouse tumor (Mouse) and SF8628 human DIPG primary cells were expanded in culture and immunostained for DAPI (nuclear), oligodendrocyte marker (Olig2, green), NG2 (red), and astrocyte marker (GFAP, magenta). Confocal microscopy was used to assess expression of above-mentioned proteins in mitotic cells. Scale bar = 5 μM.

To study the role of NG2 expression in gliomagenesis, we studied mitotically active mouse and human glioma cells. NG2 expression is typically asymmetric in non-malignant dividing stem cells, resulting in only one daughter cell ‘inheriting’ NG2 expression [13]. However, in glioma cells, NG2 expression is symmetric (inherited by both daughter cells), resulting in an increased population of uncommitted stem cells within the tumor [13]. To evaluate the NG2 expression pattern in DIPGs, we expanded human primary DIPG cells as well as neurospheres obtained from PDGFB mouse. Cells were fixed and stained using an antibody against NG2 and monitored for NG2 expression and distribution in dividing cells. Our immunocytochemical assays showed symmetric and equal expression of NG2 in both daughter cells of mouse cells and two human DIPG (SF8628, SUDIPGVI) neurospheres (Figure 4a). Symmetric NG2 expression was observed in 100% of dividing tumor cells, a characteristic previously reported in other gliomas [13].

To test the potential differentiation of NG2 expressing cells into astrocytes or oligodendrocytes *in vitro*, human primary DIPG and mouse cells were expanded in culture. Immunocytochemical assays using antibodies for oligodendrocyte (Olig2) and astrocyte (GFAP) markers were performed (Figure 4b). We detected the presence of both GFAP and Olig2 in mitotic DIPG cells that determine symmetric NG2 expression (Figure 4b). Although pediatric brainstem gliomas are historically classified as astrocytic [21, 22], these results are consistent with a recent finding that oligodendrocyte progenitors may contribute to the formation of pediatric DIPG [7]. Further analysis using flow cytometry confirmed co-expression of Olig2, GFAP and NG2 (Supplementary Figure 4). We observed that fifty percent (55.8%) of mouse neurospheres co-express Olig2 and NG2, 54% co-express NG2 and GFAP, and 55.6% co-express GFAP and Olig2 (Supplementary Figure 4). These results suggest a lack of terminal differentiation in NG2 expressing cells in DIPG.

### NG2 Expressing Neurospheres are Neoplastic *In Vivo* and result in Highly Aggressive Pontine Tumors

**Figure 5:**
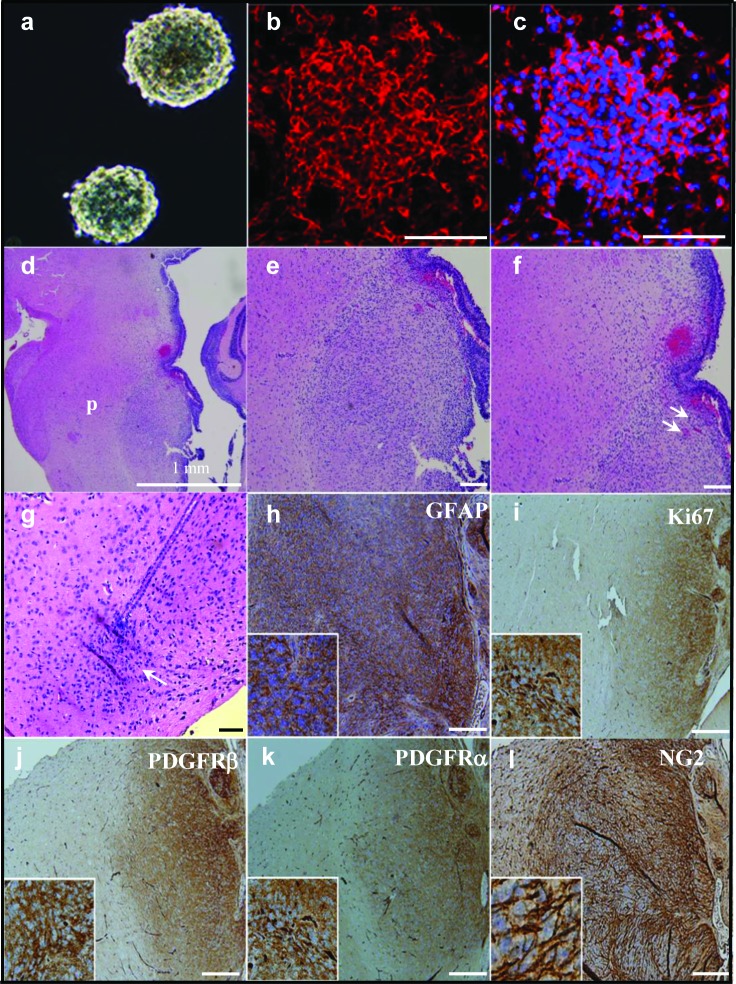
Orthotopic injection of NG2 expressing cells causes aggressive brainstem tumors *in vivo* resulting in a robust murine model of DIPG (**a**) NG2 expressing neurospheres were isolated from PDGFB mouse pontine tumor and expanded in culture and stained for NG2 (red) and DAPI (blue) (**b** and **c**). Neurospheres were then injected into the brainstems of two-day-old (P2) mice. Whole brain was obtained at 3 weeks post injection and subjected to H&E staining (**d**-**g**). Injected cells show an infiltrative pattern of growth (g, arrow) at the site of injection. H&E staining also showed a large infiltrating tumor within pons (region p in d). 10 × magnifications (**e**-**f**) revealed the highly cellular tumor within the pons (e) that exhibits characteristics of DIPGs including tumor vascularity (f, arrows). Injected cells were infiltrated through the pons and away from the injection site (g, arrow). This pontine tumor is positive for the astrocytic marker GFAP (h), proliferation marker Ki67 (i), PDGFRβ (j), PDGFRα (k), and NG2 (l). Scale bar = 100 μM.

To test the tumorigenicity of NG2 expressing cells *in vivo*, and to approximate the anatomical microenvironment of human DIPGs, we injected (100,000-150,000) NG2 expressing PDGFB mouse tumor neurospheres (Figure 5a-5c) orthotopically into the pons of 2-day old (P2) Balb/C mice and monitored for signs of tumor extension, including cerebellar ataxia [23]. Mice were euthanized at P21 and brainstems stained by H&E for analysis by a neuropathologist (Figure 5d-5g). We found that 77.8% (14 of 18) of mice had detectable, infiltrating pontine tumors by P21. When left untreated, 100% of mice died within 8 weeks post injection due to tumor growth as assessed by necropsy and examination by a neuropathologist. Histological examination of the mice brainstems showed infiltration of injected tumor cells within the pons (p) (Figure 5d), distal to the injection site (Figure 5g). The pontine tumors demonstrated histopathological characteristics of pediatric DIPGs, including hypercellularity, glial atypia, increased tumor vascularity and true microvascular proliferation (Figure 5e and 5f). Immunohistochemical analysis of tumor tissue demonstrated upregulation of NG2, Ki67 (proliferation marker), PDGFRα, PDGFRβ and GFAP (Figure 5h-5l).

### NG2 Expression is Reduced *In Vivo* Using Targeted MicroRna Strategy

**Figure 6:**
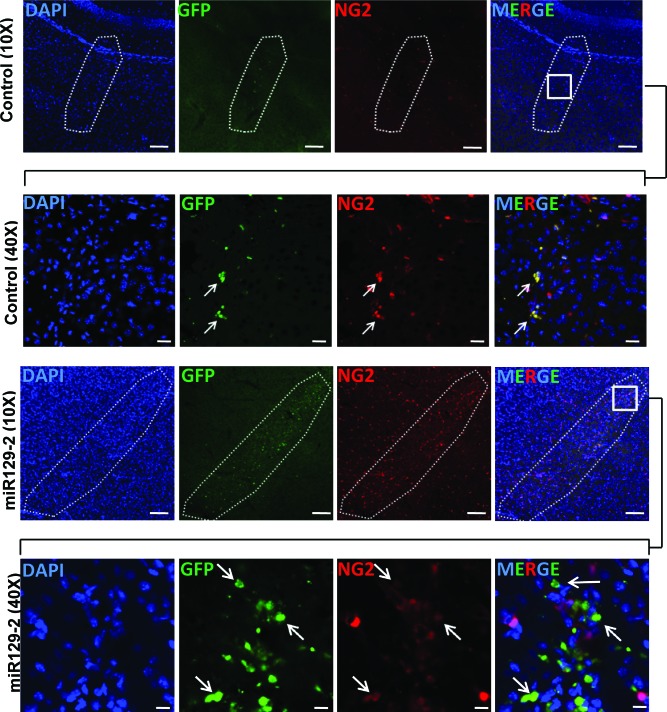
miR129-2 targets and downregulates NG2 *in vivo* Brain sections from NG2-dsRed mice were analyzed 2 weeks after the intracranial injections with control lentiviral vector on left side and lentiviral vector harboring miR129-2 on right side. Detection of the green signal (GFP) indicates *in vivo* transduction of either control or miR129-2. Area enclosed by dotted line in 10 × image (Scale bar: 100 μM) represents the site of injection. 40 × magnification images (Scale bar: 10 μM) of boxed area in merged images are shown to demonstrate the downregulation of NG2 (red) in miR129-2 (green) transduced cells (arrows) while control transduced cells are expressing normal levels of NG2.

To determine whether we can reduce NG2 expression *in vivo,* we used NG2-dsRed transgenic mice that express dsRed under the control of NG2 promoter [24]. Nine days old (P9) NG2-dsRed mice were injected orthotopically with miR129-2 and GFP expressing lentivirus on the right hemisphere, and with GFP only expressing lentivirus (Control) on contralateral hemisphere. Mice were sacrificed two weeks post-injection and brain sections were examined using confocal microscopy (Figure 6). GFP signal (green) was detected on both orthotopic injected hemispheres indicating *in vivo* transduction of lentiviral vector. Examination of the right hemisphere injected with miR129-2 lentivirus indicated non to low expression of NG2 in cells transduced by miR129-2 as judged by GFP and dsRed expression. However, in the contralateral hemisphere injected with GFP only control lentivirus, no reduction in NG2 expression was observed indicating the suitability of miR129-2 as a potential therapeutic molecule for regulating NG2 expression *in vivo* (Figure 6).

## DISCUSSION

Our study shows NG2 expression to be associated with 78% of our DIPG cohort. This observation is in line with previous measures of NG2 overexpression in a variety of cancers, including melanoma [25], glioblastoma, and lymphoid leukemia [11, 26, 27]. Moreover, NG2 expression has been shown to correlate with increased glioma malignancy [10, 28, 29]. NG2 also binds to collagen VI and laminin 2, and NG2 expressing cells migrate more effectively on collagen and laminin 2 coated surfaces compared to NG2 null cells [30]. Since collagen VI and laminin 2 are not abundant in brain parenchyma, tumor microvasculature and axonal processes may facilitate migration (and thus infiltration) of NG2 cells. Exploring NG2 and its molecular pathways may provide an opportunity for treating DIPGs by targeting factors that contribute to cellular infiltration.

Our data suggests the role of miR129-2 in regulating NG2. We show that in DIPGs, significant hypermethylation of miR129-2 promoter results in miR-129-2 downregulation. This correlates with increased NG2 mRNA and protein expression in 71.4% of studied DIPG specimens. Our study shows administration of the demethylating drug 5-Aza results in NG2 downregulation *in vitro*. 5-Aza is a DNA methyl-transferase inhibitor that has been used successfully for targeting miR129-2 in gastric cancer [18] and for epigenetic modification of *SOX2* gene in adult malignant glioma cells [19]. Given our findings that NG2 is highly expressed in a majority of pontine tumors, preclinical studies testing 5-Aza in murine models may be warranted.

Glial cells of the central nervous system are generally classified into three histologic subtypes: astrocytes, microglia, and oligodendrocytes. NG2 expressing cells represent the largest number of dividing cells in the adult brain [31]. The observation that NG2 expressing cells give rise to oligodendrocytes in the developing and mature CNS led to recognition of NG2 expressing cells as oligodendrocyte progenitor cells (OPCs). However, the likelihood of NG2 expressing cells to generate oligodendrocytes or other committed cells is highly debated (reviewed by [32]). Experiments using a tamoxifen-inducible NG2 Cre-ER reporter transgenic mouse model demonstrated anatomic- and age-dependence of NG2 cell differentiation [33]. These experiments revealed that NG2 cells of postnatal brain develop into oligodendrocytes, whereas NG2 cells in embryonic brain generate a subset of astrocytes in the ventral forebrain in addition to oligodendrocytes. Our observation that NG2 positive cells co-express oligodendrocyte (Olig2) and astrocyte (GFAP) cell markers may suggest their pluripotent potential. However, GFAP expression does not always indicate astrocytic lineage since introduction of the transcription factor NF1A has been shown to induce GFAP expression and astrocytic phenotype in OPC-derived tumors [34]. Although NG2 expressing cells differentiate into astrocytes or oligodendrocytes depending on age and neuroanatomical location [33], terminal differentiation of these cells may be in disarray in DIPG. This dysregulation of cell differentiation may result in a population of pluripotent tumor progenitor cells leading to a highly infiltrative and lethal cancer. Using human and PDGFB mouse DIPG cells, we demonstrated dichotomous Olig2 and GFAP expression, which may indicate the uncommitted pluripotent state of these cells. However, our results are consistent with others suggesting oligodendrocyte lineage precursors as cells of origin for DIPGs [7, 35, 36].

Asymmetric cell division plays an important role in organism development by generating committed progeny while at the same time maintaining a pool of progenitor stem cells to facilitate further development. Recently, NG2 expressing oligodendrocytes, which are glioma tumor progenitors, have been shown to exhibit defective asymmetric cell division [13]. This abnormal, symmetric cell division results in over-population of oligodendroglial precursor cells with impaired terminal differentiation, leading to neoplasia. Our observation of symmetric division in NG2 expressing mouse tumor neurospheres as well as human DIPG primary cells is consistent with these findings. Further studies are required to assess the expansion of NG2 expressing cells in DIPGs and their role in tumor progression.

We show that orthotopic injection of (150,000) NG2 expressing mouse cells into pons of P2 mice results in generation of a highly proliferative and infiltrative pontine tumor. More importantly, we demonstrate that this mouse model exhibits a short latency to tumor development (77.8% at three weeks post injection) making it representative of the aggressive clinical course of DIPG, and suitable for rapid testing in preclinical studies. Other published murine models of brainstem tumors have used similar number of cells (100,000-200,000 cells) for intracranial injection [7, 16, 37]. Therefore, the number of injected cells is not contributing to the short latency. Due to its anatomic location and the difficulty in obtaining tissue for molecular study, DIPG is historically an understudied pediatric tumor. The NG2 murine model can be complementary to existing genetically engineered [16] and the xenograft [7] models to further characterize DIPG biology and advance towards improved diagnosis and treatment. Furthermore, we show that *in vivo* targeting of NG2 by miR129-2 results in downregulation of NG2, providing a strong candidate for targeted therapy for regulating NG2 expression.

## MATERIALS AND METHODS

### Human tissue specimens

Human DIPG specimens were obtained postmortem [38] in accordance with Institutional Review Board approvals (#4932; #3804). Patient identifiers were removed prior to evaluation, and a single sequential numerical identifier was assigned to each patient (Supplementary Table 1). All brain tumor diagnoses were made radiographically by a neuroradiologist.

### Mice

Balb/C, heterozygous J:Nu, NG2-dsRed, and NOD *SCID* gamma mice were purchased (JAX Mice and services) and used in accordance with approved institutional animal use and care protocols (IACUC #292-12-05, #01429, #01335). Frozen murine brainstem glioma specimens were obtained from Oren Becher [16] and processed as described below.

### Western blot analysis

Western blotting was performed as described before [8]. Briefly, tissue lysates were prepared in 1X SDS Laemmli sample buffer containing 1 mM DTT and protease inhibitors. After protein concentration determined, 20 μg protein from each sample was resolved on 4-12% gels by SDS-PAGE. Proteins were then electroblotted onto nitrocellulose membranes and probed with appropriate primary antibody for 1 h at room temperature. After incubation with corresponding HRP-conjugated secondary antibodies, membranes were washed and antigens were detected by autoradiography using an ECL kit.

### Antibodies

The following dilutions were used: NG2-rabbit polyclonal antibody (EMD Millipore) at 1:1000 (Western), 1:200 (immunofluorescence/immunohistochemistry) and 1:100 (flow cytometry); PDGFRα at 1:500, PDGFRβ at 1:50 (Cell Signaling Technology); Ki67 (Abcam) at 1:200; GFAP-chicken polyclonal antibody (Abcam) at 1:1000 (immunofluorescence) and 1:100 (flow cytometry), Olig2-mouse monoclonal (EMD Millipore) at 1:200 (immunofluorescence) and 1:100 (flow cytometry). HRP secondary antibodies (Kirkergaard and Perry laboratories) at 1:5000 dilution for western blotting and 1:200 dilution for immunohistochemistry, all Alexa labeled antibodies (Life Technologies) at 1:500 dilution. For flow cytometry, 1 μg of Alexa 488 or Alexa 647 labeled antibodies and 0.25 μg of PE cy 5.5 in 100 μl of sample was used.

### Immunohistochemistry

Immunohistochemical analysis was performed on formalin fixed paraffin-embedded tissue sections as previously described [8]. Briefly, slides were first de-paraffinized and blocked in 1% BSA. Primary binding was performed by incubating the slides for 1 h with appropriate primary antibodies diluted in blocking buffer. Antigen levels were then detected by incubation with HRP labeled secondary antibodies followed by DAB assay.

### Cell culture

PDGFB and PDGFB/H3.3-K27M/p53^−/−^ mouse neurospheres were grown from tumor tissue obtained from mouse pontine tumors [16, 17]. Cells were dissociated and expanded in culture in the presence of mouse NeuroCult basal media supplemented with 10% mouse cell proliferation supplement (Stem Cell Technologies), glutamine, penicillin-streptomycin, heparin (2 μg/mL), rm EGF (20 ng/mL) and rm FGF (10 ng/mL). PDGFB mouse monolayer adherent cells were developed by culturing neurospheres in DMEM media supplemented with 10% FBS, glutamine, and antibiotics. Human DIPG primary cells (SF8628) were obtained from Dr. Nalin Gupta [39]. SUDIPGIV and SUDIPGVI were obtained from Dr. Michelle Monje and cultured according to already established protocol [7]. HEK 293FT cells (Life Technologies) were commercially obtained. SF8628 and HEK 293FT cells were cultured in DMEM supplemented with 10% FBS, glutamine, and antibiotics. Treatment of cells with 5-Azacytidine (Sigma) was performed by adding 1000 × solution, freshly prepared in media.

### Luciferase assay

PDGFB mouse neurospheres were seeded into 6-well tissue culture plates (200,000/well) in complete media and maintained at 37^o^C for 24 h. The cells were then co-transfected in triplicates with empty vector or miR129-2(5′ CUUUUUGCGGUCUGGGCUUGC 3′) plasmid (Origene) and luciferase reporter construct (Origene) harboring 3′ UTR of NG2 downstream of luciferase gene, using XtremeGene HP transfection reagent (Roche). Control transfections were performed by transfecting cells with miR129-2 plasmid alone or luciferase reporter construct alone. After 72 h, cell lysates were prepared and luciferase expression was determined using luciferase assay kit according to the manufacturer's protocol (Promega).

### NG2 knockdown with shRNA and miR129-2

PDGFB mouse neurospheres and adherent mouse tumor cells were transfected with NG2 specific shRNA (Origene) or control shRNA or miR129-2 plasmids or empty vector using XtremeGene HP transfection reagent, according to the manufacturer's protocol (Roche). For stable transfection in adherent mouse tumor cells, media was replaced 48 h post-transfection with fresh media containing 0.5 μg/ml of Puromycin (Sigma) and selected for NG2 knock down by changing the puromycin containing media every 2 days. Stable cells for NG2 knock down were collected and cultured starting from the day when untransfected cells showed 100% cell death in presence of puromycin.

### Construction of miR129-2 lentiviral vector

Cloning of precursor sequence of miR 129-2 (5′UGCCUUUCGCGAAUCUUUUU CUGUACAUAACUCAAUAGCCGGAAGCCCUUACC CCAAAAAGCAUUCGCGGAGGGCG 3′) into HIV based lentiviral vector, pCDH-EF1-MCS-BGH-PGK-GFP-T2A-Puro vector (System Biosciences), was performed by GenScript. Briefly, miR129-2 precursor sequence from pmiR129-2 plasmid was isolated and cloned into pCDH-EF1-MCS-BGH-PGK-GFP-T2A-Puro vector using *EcoR1* and *Not1* restriction sites. After sequence verification, *E.coli* were transformed with the right clone and vector was expanded by DNA mini-preparation.

### Production of lentivirus

Plasmids for 3rd generation lentiviral packaging system (pMDLg/pRRE, pRSV-Rev, pMD2.G) were purchased from Addgene. Six million HEK293FT cells were plated into T75 cell culture flasks 24 h before transfection. Using XtremeGene HP transfection reagent (60 μl), cells were co-transfected with 10 μg of control-pCDH or pCDH-miR129-2 vectors and packaging plasmids. A total of 10 μg of DNA was used for packaging plasmids (**5** μg **of pMDLg/pRRE and 2.5** μg **each of pRSV-Rev and pMD2.G).** After overnight incubation, media was replaced with fresh media and maintained under normal growth conditions. Virus containing supernatant was collected after 24 h and fresh media was added to cells, which was collected again as virus after additional 24 h incubation. Viral supernatants were then concentrated using Millipore Amicon Ultracel 100K spin columns and titered using QuickTiter Lentivirus Titer Kit (Cell Biolabs).

### Lentiviral transduction of human DIPG primary cells

SF8628 cells were seeded into 6 well cell culture plates and maintained for 24 h under normal growth conditions. Media was then replaced with 2 ml of fresh media containing 5 μg/ml of polybrene and added 0.4 million TU in 20 μl of control pCDH or pCDH miR129-2 vectors to cells. 24 h post-transduction, virus containing media was replaced with fresh media and maintained under normal growth conditions for 48 h.

### *In vivo* lentiviral transduction

NG2-dsRed pups of day P9 were anesthetized by inducing hypothermia. Injection area was sterilized and exact injection spot was marked 2 mm right or left to the sagittal suture and 1 mm posterior to the bregma. Using a 26-gauge needle fitted to a gas tight Hamilton syringe, 2 μl (2×10^8^ TU/ml) of pCDH-miR129-2 lentiviral vector was injected on the right side and pCDH control vector was injected on the left side of the same mouse. After recovery, animals were returned to their home cages and monitored daily.

### Immunofluorescence

Cells grown on poly-l-lysine and laminin coated chamber slides were washed with PBS, fixed in 10% neutral buffered formalin solution for 15 min, and permeabilized in 0.1% Triton X-100 for an additional 10 min. Cells were then blocked in PBS containing 1% BSA for 1 h, followed by the incubation with primary and fluorescent tagged secondary antibodies. After washing in PBS, cells were briefly stained with 4′,6-Diamidino-2-phenylindole (DAPI) and imaged using laser scanning confocal microscope (Zeiss) and ZEN 2009 software (Zeiss).

### Scratch assay

Adherent PDGFB mouse tumor cells were subjected to one of four experimental arms: NG2 knock-down with shRNA, transient transfection with mirR129-2, transfection with control shRNA, no treatment or empty vector. All treatment groups were serum starved for 24 h. Cell monolayer was then scraped in a straight line with a p200 pipet tip and washed with PBS to rinse away any floating cells. Control cells were then treated with either vehicle (media) or 10 μM 5-Azacytidine and imaged using 10 × objective at 0 and 24 h time intervals and scratch area measured using image J software. Percentage cell invasion was calculated as follows: Percent invasion = (A_t = 0 h_ − A_t = Δ h_)/A_t = 0 h_ × 100%, as described previously [40], where, A_t = 0 h_ is the area of the invasion measured immediately after scratching and A_t = Δ h_ is the cell invasion area measured at 24 h after scratching.

### FACS analysis

Cells were fixed with 2.5% neutral buffered formalin and permeabilized in 90% ice-cold methanol for 30 min on ice, washed and blocked using FACS buffer (0.5% BSA in PBS). A mixture of GFAP, NG2 and Olig2 antibodies diluted in FACS buffer was added to cells and incubated for 1 h at room temperature, washed, and incubated for 30 min in fluorescent labeled secondary antibody mixture. Cells were then washed and re-suspended in FACS buffer and loaded into a FACS Calibur (BD Biosciences) followed by analysis using Flowjo software (Tree Star).

### Orthotopic injection of mouse neurospheres

Pups (P2) were used for intracranial injection. Area of injection was sterilized and a total of 150,000 live tumor cells in 2 μl volume were injected into the brainstem (2 mm posterior to the bregma at the midline position) of cold anesthetized pups using a 26-gauge needle fitted to a gas tight Hamilton syringe [16]. NG2 expressing cells were generated by expanding cells obtained from PDGFB expressing brainstem glioma mouse model [16].

### QRT-PCR for miR129-2 expression

TaqMan small RNA assays kit (Applied Biosystems) was used to quantify the expression of miR129-2 in human DIPG samples. Briefly, 10 ng of total RNA in a final volume of 15 μl including 100 mM dNTPs, 50U of Multiscribe Reverse Transcriptase, 5 × RT primer (provided in the kit) was incubated at 16°C for 30 min, 42°C for 30 min followed by enzyme inactivation at 85°C for 5 min. QPCR of miR129-2 was performed according to manufacturer's instructions. The reaction was done in 20 μl total volume with 1.33 μl of cDNA, 1 μl of TaqMan small RNA assay (20 ×), and 10 μl of TaqMan Universal PCR Master Mix. QPCR of RNU48 gene was used as a housekeeping control. Fold changes were obtained using comparative C_T_ method.

## SUPPLEMENTARY MATERIAL FIGURES AND TABLES


