# Supplementary material for: The emerging role of NG2 in pediatric diffuse intrinsic pontine glioma
Source: Oncotarget. 2015 Mar 30;6(14):12141–55. doi: 10.18632/oncotarget.3716 (PMC4494928; doi:10.18632/oncotarget.3716)
Supplement: Supplementary file 1 [file oncotarget-06-12141-s001.pdf]

The emerging role of NG2 in pediatric diffuse intrinsic pontine glioma

Supplementary Material

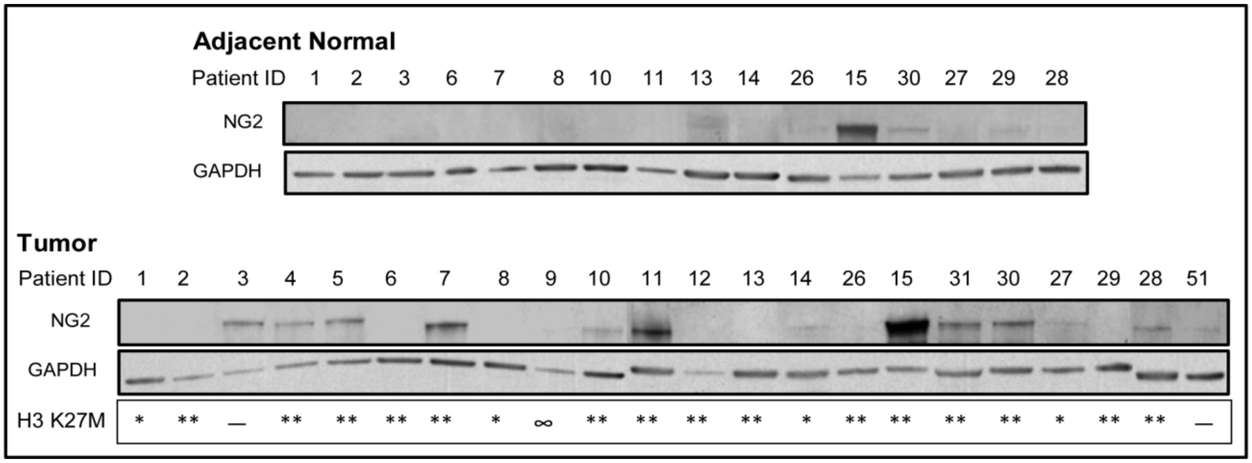

Supplementary Figure 1: Western blot analysis of NG2 in human DIPG tumor and adjacent normal tissues and status of H3 mutation. Western blot analysis validated NG2 expression in 13/22 (59%) of DIPG specimens tested. Adjacent normal specimens (n=16) were used as controls. Low NG2 expression was detectable in 4 normal specimens possibly due to infiltration of NG2 expressing cells. GAPDH was used for protein loading normalization. Histone 3 mutation status is given for histone 3.1 K27M mutation (*), histone 3.3 K27M (**) or wild type (∞). NG2 expression was detected in 100% of patients with H3 K27M mutations.

a

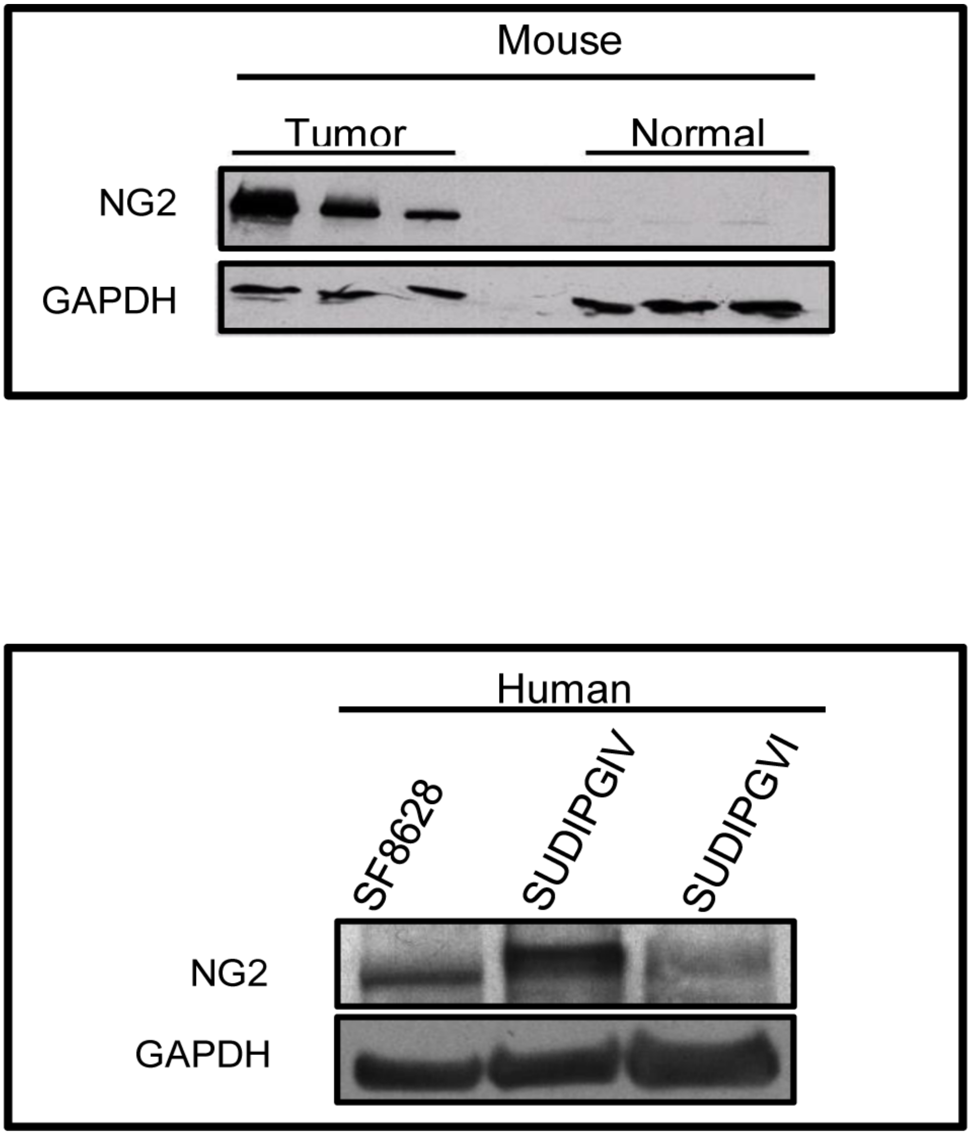

Supplementary Figure 2: NG2 expression in PDGFB mouse tumor and human DIPG primary cells. (a) Western blotting was performed using mouse normal and tumor tissues. High expression level of NG2 was detected in protein extracts from PDGFB mice (n=3) tumors compared to healthy pons (n=3) from age-matched mice. GAPDH was used for protein loading normalization. (b) NG2 expression is detected in three primary human DIPG cells.

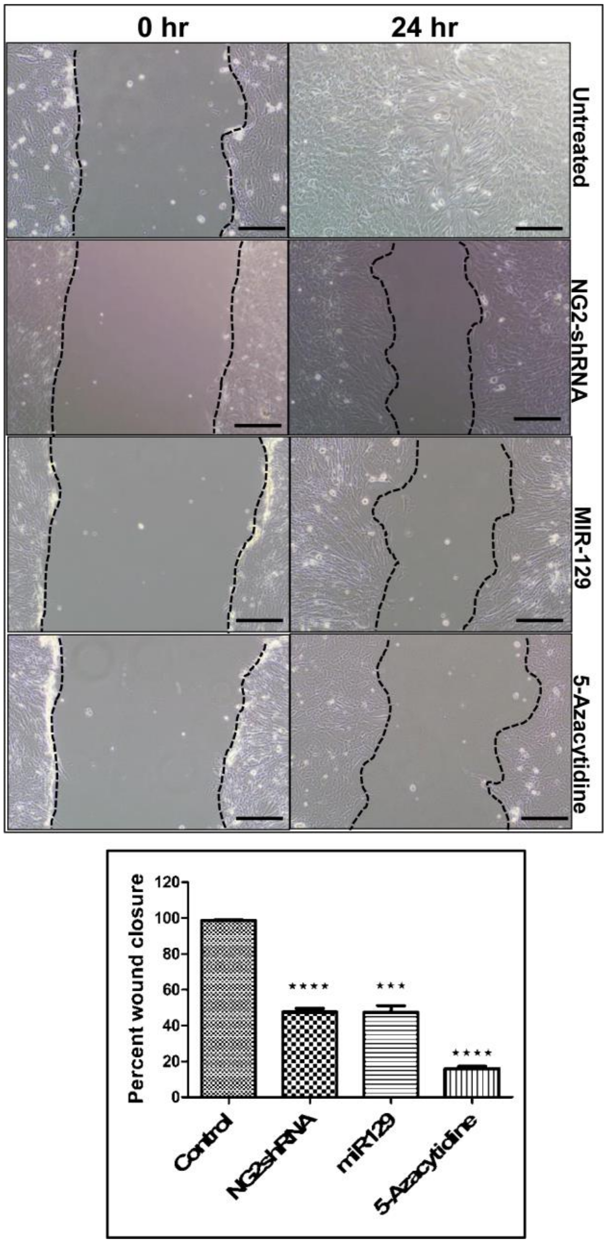

Supplementary Figure 3: NG2 inhibition retards cellular migration *in vitro*. To assess the role of NG2 in cellular migration, PDGFB adherent mouse tumor cells were expanded in culture and transfected with NG2-shRNA (stable), miR129-2 (transient) or treated with 5-Azacytidine. Scratch wound assay was performed and wound area was imaged and measured at 0 h and 24 h time intervals. Scale bar = 200 µm. Quantitation of percent wound closure at 24 h as compared to 0 h (bottom panel).

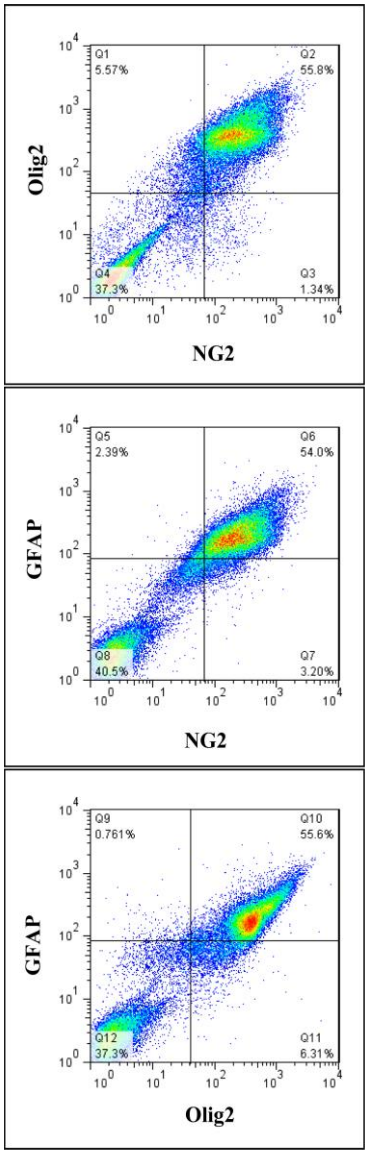

Supplementary Figure 4: NG2 expressing cells lack terminal differentiation. Primary neurospheres from PDGFB mouse tumor were fixed and immunostained using primary antibodies for NG2, GFAP, and Olig2 for flow cytometry analysis. Mouse primary cells co-express NG2 and Olig2 at 55.6%. Similarly, 54% of mouse neurospheres co-express both GFAP and NG2. Co-expression of astrocyte (GFAP) and oligodendrocyte marker (Olig2) is 55.6%. The robust co-expression of any two antibodies indicates the lesser degree of commitment of mouse tumor cells.

Supplementary Table 1: Human postmortem DIPG specimens used for IHC and molecular analysis. Missing information is indicated as n/a (not available).

Patient ID	Diagnosis	Gender	Age	H3.1 / H3.3 Mutation status	Tumor	Normal
1	DIPG	M	8y	H3.1K27M	Brainstem	Brainstem
2	DIPG	F	6y	H3.3K27M	Brainstem	Brainstem
3	DIPG	F	7y	n/a	Brainstem	Brainstem
4	DIPG	M	8y	H3.3K27M	Brainstem	n/a
5	DIPG	M	n/a	H3.3K27M	Brainstem	n/a
6	DIPG	F	14y	H3.3K27M	Brainstem	Brainstem
7	DIPG	F	5y	H3.3K27M	Brainstem	Brainstem
8	DIPG	M	n/a	H3.1K27M	Brainstem	Brainstem
9	DIPG	F	4y	WT	Brainstem	n/a
10	DIPG	M	8y	H3.3K27M	Brainstem	Brainstem
11	DIPG	M	8y	H3.3K27M	Brainstem	Brainstem
12	DIPG	n/a	n/a	H3.3K27M	Brainstem	n/a
13	DIPG	M	21y	H3.3K27M	Brainstem	Frontal Lobe
14	DIPG	M	6y	H3.1K27M	Brainstem	Frontal lobe
15	DIPG	F	9y	H3.3K27M	Brainstem	Cerebellum
16 through 25	DIPG	n/a	n/a	n/a	Brainstem	-
26	DIPG	F	9y9m	H3.3K27M	Brainstem	Parietal lobe
27	DIPG	M	5y6m	H3.1K27M	Brainstem	Frontal

**28**	**DIPG**	**M**	**4y**	**H3.3K27M**	**Brainstem**	**Frontal Lobe**
**29**	**DIPG**	**M**	**8y**	**H3.3K27M**	**Brainstem**	**Frontal**
**30**	**DIPG**	**F**	**10y**	**H3.3K27M**	**Brainstem**	**Frontal Lobe**
**31**	**DIPG**	**n/a**	**12y**	**H3.3K27M**	**Brainstem**	**n/a**
**32-33**	**DIPG**	**n/a**	**n/a**	**n/a**	**Brainstem**	**-**
**51**	**DIPG**	**F**	**7y**	**H3.3K27M**	**Brainstem**	**n/a**

Supplementary Table 2: Immunohistochemical assays assessing NG2 and PDGFR-α expression in formalin fixed DIPG specimens. NG2 and PDGFR-α staining were done by the automated immunohistochemistry Ventana Medical Systems and read by a pathologist. The rating parameters were as follows: (Negative): No membranous staining was seen; (+0.5): weak membranous staining was seen; (+1): moderate membranous staining was seen; (+2): strong membranous staining was seen.

**Specimen ID**	**PDGFR-α**	**NG2**
	**Rating**	**Rating**
**16**	+1	Negative
**17**	+2	+1
**18**	N/A	+2
**19**	Negative	Negative
**20**	+2	Negative
**21**	Negative	+1
**22**	+1	+1
**23**	+2	+0.5
**24**	Negative	+1
**25**	+2	+2
**32**	N/A	+1
**33**	N/A	+2
